# Study on the 1990–2021 trend of global childhood respiratory infection and tuberculosis disease burden and related risk factors

**DOI:** 10.3389/fpubh.2025.1609990

**Published:** 2025-07-30

**Authors:** Jie Chen, Chao Fang, Weihong Lu, Xiangtao Wu, Xingliang Zhang

**Affiliations:** ^1^Department of Medical Oncology, Affiliated Hengyang Hospital of Hunan Normal University & Hengyang Central Hospital, Hengyang, China; ^2^Department of Pediatrics, Affiliated Hengyang Hospital of Hunan Normal University & Hengyang Central Hospital, Hengyang, China; ^3^Department of Pediatrics, The First Affiliated Hospital of Xinxiang Medical University, Xinxiang, China; ^4^Department of Respiratory Medicine, Institute of Pediatrics, Shenzhen Children's Hospital, Shenzhen, China

**Keywords:** global, children, respiratory tract infection, tuberculosis, disease burden, risk factors

## Abstract

**Background:**

Respiratory tract infections (RTIs) and tuberculosis (TB) impose a critical global health burden on children, serving as leading causes of morbidity and mortality. Lower respiratory tract infections (LRIs) remain the primary cause of death in under-5 s, though mortality has declined recently.

**Objective:**

This study aims to analyze trends in RTIs and TB among 0–14-year-olds using Global Burden of Disease (GBD) data from 1990 to 2021.

**Methods:**

Global data on childhood RTIs and TB were collected from GBD, with standardized methods used to assess disease burden trends, age/sex/SDI differences, and the contribution of 11 risk factors.

**Results:**

From 1990 to 2021, incidences of upper RTIs, otitis media, and TB decreased, but overall RTIs increased. Neonatal LRI had the highest mortality (1,560.6/100 k). Male children showed higher TB incidence/mortality. Low-SDI areas had the highest burden (mortality 2.036/100 k), while high-SDI areas saw the largest TB mortality drop (95.7%). Underweight remained the main risk factor, with DALY rate falling 80.3%, though household air pollution, low birth weight, short gestation, and high temperature rose in rank.

**Conclusion:**

Global childhood respiratory disease burden faces challenges, requiring strengthened international cooperation and targeted interventions, especially in low-SDI regions, to improve public health and nutrition.

## Introduction

Lower respiratory tract infections (LRIs) and tuberculosis (TB) remain the leading causes of childhood morbidity and mortality, with the former long having been the leading cause of death in children under 5 years of age, although the mortality rate has declined considerably in recent years (1–3). The global burden of LRIs has declined due to improved public health interventions (3). However, TB remains a major global health problem, with approximately 239,000 children dying from the disease in 2015, the vast majority of whom did not receive timely treatment (4). The burden of childhood TB is particularly heavy in areas with high TB incidence, such as Southeast Asia and Africa (4, 5).

Malnutrition, especially childhood wasting, is one of the most important risk factors for LRI-related mortality (1). It also significantly increases the risk of TB in children and accelerates the progression of the disease (4). Globally, approximately 45 million children under the age of 5 are wasted, with these children mainly concentrated in low-and middle-income countries (6). In addition, indoor air pollution, exposure to second-hand smoke, and poor living conditions are important risk factors for LRIs and TB, with children exposed to second-hand smoke having a higher risk of the latter (6). Second-hand smoke exposure is more common in low-socio-demographic index (SDI) areas, and the risk of TB in children exposed to second-hand smoke is several times that of unexposed children (7). Studies have shown that socioeconomic status has a profound impact on the incidence and outcomes of childhood respiratory tract infections (RTIs) and TB, and low-income areas bear a heavier disease burden (3, 6).

Although the global burden of childhood RTIs and TB has declined, especially in terms of mortality – largely due to vaccination, public health interventions and improved health awareness – there are marked variations in risk factor impact across different SDI regions. In high-SDI areas, the morbidity and mortality of RTIs and TB in children are relatively low due to abundant medical resources, good sanitary conditions and improved public health policies (8). However, in low-SDI areas, children are more vulnerable to these diseases due to the lack of basic medical facilities, serious malnutrition problems and inadequate public health measures (7).

Globally, public health policies play an important role in the prevention and control of RTIs and TB in children. The ‘End Tuberculosis Strategy’ issued by the World Health Organisation (WHO) aims to reduce TB mortality by 90% and TB incidence by 80% by 2030 compared with 2015, and sets the additional goal of further reducing mortality and incidence by 2035 (9). In the future, the key to reducing the burden of RTIs and TB in children in low-income areas lies in addressing the underlying risk factors, such as malnutrition, air pollution and socioeconomic inequality. Only by further strengthening global public health interventions, improving the allocation of medical resources and enhancing socioeconomic status can the disease burden in these areas be effectively reduced.

## Materials and methods

### Study design

This study adheres to disease classifications defined by the Global Burden of Disease (GBD) 2021 study. According to GBD criteria, upper respiratory tract infections (URIs) include nasopharyngitis, sinusitis and tonsillitis (ICD-10 codes J00–J06); LRIs include pneumonia (J12–J18) and bronchitis (J40–J42), with neonatal LRIs specified as infections within 28 days of birth; otitis media is categorised under ear infections (H65–H67), including acute and chronic cases; and TB includes active pulmonary TB (A15–A16) and tuberculous pleurisy (A17.0). Here, all diseases were standardised using GBD’s coding system to ensure comparability.

Using GBD 2021 data, we performed cross-sectional trend analysis on age-, sex-and SDI-stratified burden (incidence, mortality) in 204 countries (1990–2021). The SDI categorises regions by income, education and fertility, and is used to measure the level of socioeconomic development in different regions. The GBD 2021 study divides the world into five SDI levels. This study focused on children aged 0–14 years, further subdivided into three age groups to analyse the differences in disease burden among children of different ages. The data was obtained from the GBD 2021 database, which covers population health data from 204 countries and regions around the world. The sample size was determined by the GBD research team based on the census, health statistics and epidemiological survey data of various countries to ensure the representativeness and reliability of the data. The study quantified the contribution of 11 key risk factors to the disease burden, including underweight children, household air pollution (HAP), low birth weight, short pregnancy and high temperature.

The selection of risk factors was based on the risk factor framework of GBD 2021, and their contribution to disability-adjusted life year (DALY) rate was calculated through attribution analysis. Here, the standardised attribution analysis method in GBD research was used to calculate the population attributable fraction (PAF) of each risk factor, and combined with the exposure level and relative risk (RR) value to evaluate its contribution to the disease burden. In addition, a multivariate regression model was used to analyse the relationship between risk factors and disease burden, and confounding variables were controlled to determine independent effects.

### Data sources

This study extracted epidemiological data on RTIs and TB in children aged 0–14 years in 204 countries and regions around the world from the 2021 GBD database, including age-, sex-and SDI-stratified data on incidence, mortality and DALY rate. In addition, risk factor-related data included PAF, exposure level and RR. The SDI indicator divides countries/regions into five categories (low, medium-low, medium, medium-high and high SDI) based on per capita income, education level and fertility rate. Only model-corrected data officially released by GBD were included. The GBD 2021 dataset was processed through age-, sex-and SDI-stratified screening for 204 countries/regions, excluding indicators with >10% missing values, followed by spatial interpolation and Bayesian hierarchical modelling (incorporating spatial autocorrelation inference from neighbouring regions) to address data scarcity, with multi-source evidence integrated via meta-analysis. Cross-validation against national statistics and WHO reports constrained errors within 95% confidence intervals (CIs) (detailed in Supplementary Table 1), with technical details following GBD 2021 methodology reports (1, 2).

### Data collection and processing

First, relevant datasets were downloaded from the GBD database and preliminarily screened. Subsequently, data cleaning was performed for missing values and outliers, and missing data were filled using interpolation methods to ensure data integrity and consistency. Statistical software, R language (IBM, Armonk, NY, United States) was used for data verification to ensure data accuracy and reliability. All data were standardised before analysis to eliminate deviations in different regions and time periods.

### Study population

This study divided the world’s children aged 0–14 into three groups according to age: infants and toddlers (0–4 years old), preschoolers (5–9 years old) and early adolescents (10–14 years old). The exclusion criteria included individuals aged ≥15 years, countries/regions with >10% missing data (12 countries excluded, 5.9% of global total) and regions with distorted data due to war/natural disasters (e.g., Syria post-2011, Yemen 2020; four countries in total). Finally, data from 190 countries/regions were included, covering 94.1% of the global child population.

### Risk factors

The included risk factors were selected based on the GBD comparative risk assessment framework, involving 11 major factors related to childhood RTIs and TB: malnutrition (low weight, stunted growth), HAP (solid fuel use), ambient particulate matter pollution (PM2.5), second-hand smoke exposure, insufficient breastfeeding, HIV infection, insufficient vaccine coverage (e.g., pneumococcal vaccine), crowded living, lack of sanitation, zinc deficiency and vitamin A deficiency. The contribution of each risk factor to DALY rate was quantified by the PAF:


$$\mathrm{PAF}=\frac{\sum_{i}p_{i}({\mathrm{RR}}_{i}-1)}{\sum_{i}p_{i}({\mathrm{RR}}_{i}-1)+1}$$

where *pi* represents the proportion of people exposed at the *ith* exposure level and *RR_i_* represents the relative risk at the *ith* exposure level. The RR value was based on the risk factor-disease association data provided by the GBD 2021 database and was corrected in combination with the results of the meta-analysis. The exposure level data was obtained from the GBD 2021 exposure database, including age-, gender-and SDI-stratified data for 204 countries and regions around the world.

### Data analysis

The age-standardised rate (ASR) was calculated using the GBD standard population structure to eliminate the impact of differences in age distribution in different regions on morbidity and mortality. Joinpoint regression was used for time-trend analysis to identify significant change nodes (turning years) of disease burden indicators between 1990 and 2021, and the estimated annual percentage change rate (EAPC) and average annual percentage change rate were calculated. Linear mixed models were used for SDI-stratified comparison to assess the convergence or divergence of trends between different SDI groups. Gender and age differences were analysed using the sex incidence rate ratio (IRR = male/female incidence rate) and mortality rate ratio, and the significance of the differences was assessed using the chi-square test. Multilevel models were used to analyse the impact of age subgroups on disease burden. Risk factor attribution analysis used the PAF formula to quantify the contribution of each risk factor to the disease burden. Segmented regression analysis was used to assess the annual rate of change of PAF and identify key drivers. Sensitivity analysis was performed using the 95% uncertainty interval (UI) provided by GBD, and the robustness of the results was verified by 1,000 Monte Carlo simulations. In addition, years with extreme events (e.g., data during the COVID-19 pandemic) were excluded to test the stability of the trend. All data were processed for missing values, and interpolation methods were used to fill in missing data to ensure data integrity. Trend assessment compared differences between different populations by ASRs, calculated EAPCs and used R language software (version 4.2.0) for missing value interpolation and multivariate regression analysis.

## Results

### Global incidence of respiratory tract infections and tuberculosis in children aged 0–14 years in 2021

**Figure 1:**
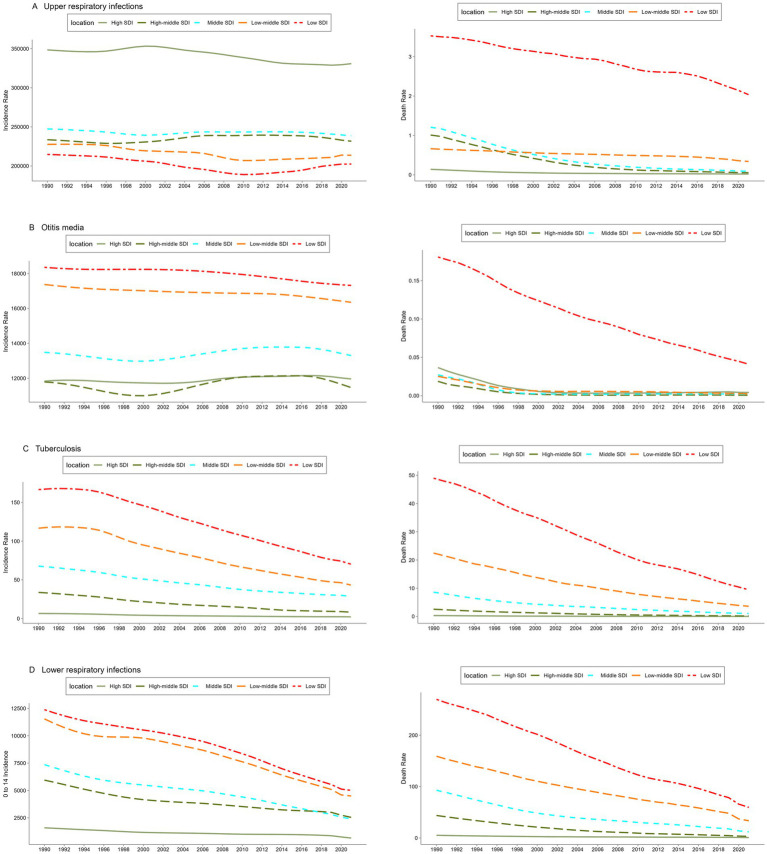
Trends in the incidence and mortality of upper respiratory tract infections. **(A)** Otitis media, **(B)** tuberculosis, **(C)** lower respiratory tract infections, and **(D)** among children aged 0–14 years in the world and in different SDI regions from 1990 to 2021. SDI: Socio-demographic index.

**Figure 2:**
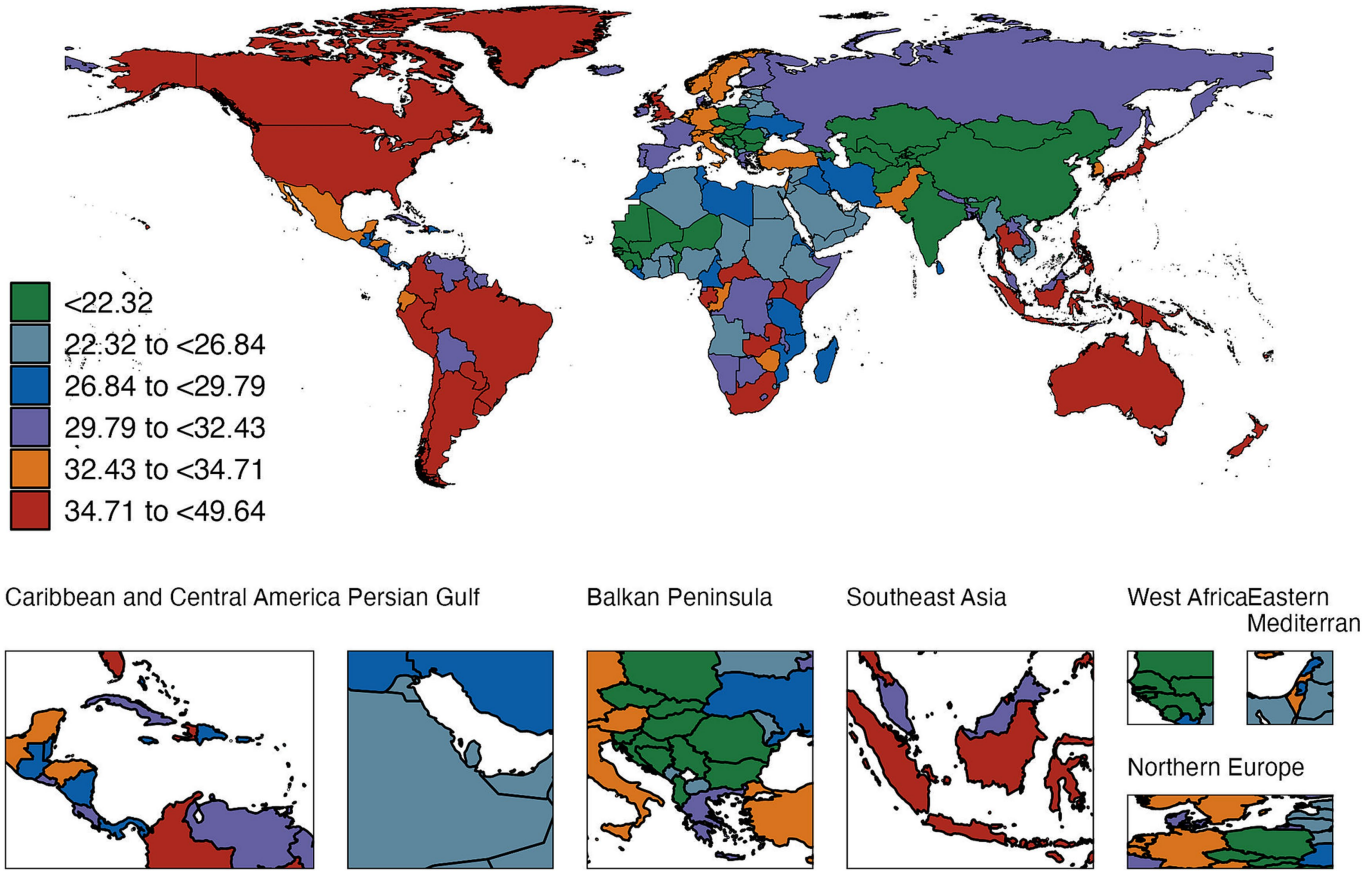
Global incidence of RTIs and TB in children (0–14 years) in 2021 (per 1,000,000 population; error bars: 95% UI).

In 2021, the total incidence of RTIs and TB in children aged 0–14 years worldwide was 558.536 million cases (95% CI: 468.510, 660.197), of which TB accounted for approximately 0.007 million cases (95% CI: 0.005, 0.009), URIs for 463.397 million cases (95% CI: 371.762, 565.886), LRIs for 6.990 million cases (95% CI: 6.213, 7.979) and otitis media for 29.724 million cases (95% CI: 20.520, 43.173). While the incidence of URIs, otitis media and TB decreased, the age-standardised overall incidence of RTIs (excluding TB) rose from 269.47/100 k in 1990 to 277.62/100 k in 2021 (EAPC = 0.124%; 95% UI: 0.081–0.166), reflecting a shift in disease composition rather than population growth (Table 1). This paradox is driven by diagnostic disparities across SDI regions: high-SDI areas, with the highest URI incidence but lowest mortality (Figure 1A), likely detect more mild cases via advanced diagnostics (e.g., PCR), whereas low-SDI regions – with the highest TB mortality (Figure 1C) – underreport non-severe infections, skewing global trends. In 2021, the global overall incidence of RTIs and TB in children was 277.62/100 k (269.47/100 k in 1990), with an EAPC of 0.124% (95% UI: 0.081–0.166). Notably, TB incidence declined dramatically (EAPC = −3.9%), as did LRI incidence (EAPC = −2.8%), whereas URI incidence remained stable (EAPC = 0.02%). The global overall incidence of RTIs and TB in children was 277.62/100 k (269.47/100 k in 1990), with an EAPC of 0.124% (95% UI: 0.081–0.166) (Figure 2).

**Table 1:** Key global burden trends of childhood RTIs and TB (1990–2021).

Indicator	1990	2021	EAPC (%)	95% UI
Overall RTI/TB incidence	532.1 million cases	558.5 million cases	+0.12	0.08–0.16
Age-standardized RTI rate	269.47/100,000	277.62/100,000	+0.124	0.081–0.166
Neonatal LRI mortality	1,560.6/100,000	1,210.3/100,000	−0.78	−0.85 – −0.71
Low-SDI TB mortality	2.036/100,000	1.452/100,000	−1.02	−1.15 – −0.89

EAPC: Estimated annual percentage change, SDI: Socio-demographic Index; UI: Uncertainty interval.

### Number of cases and deaths of respiratory tract infection-related diseases in children worldwide in 2021, and age-specific morbidity and mortality rates

**Figure 3:**
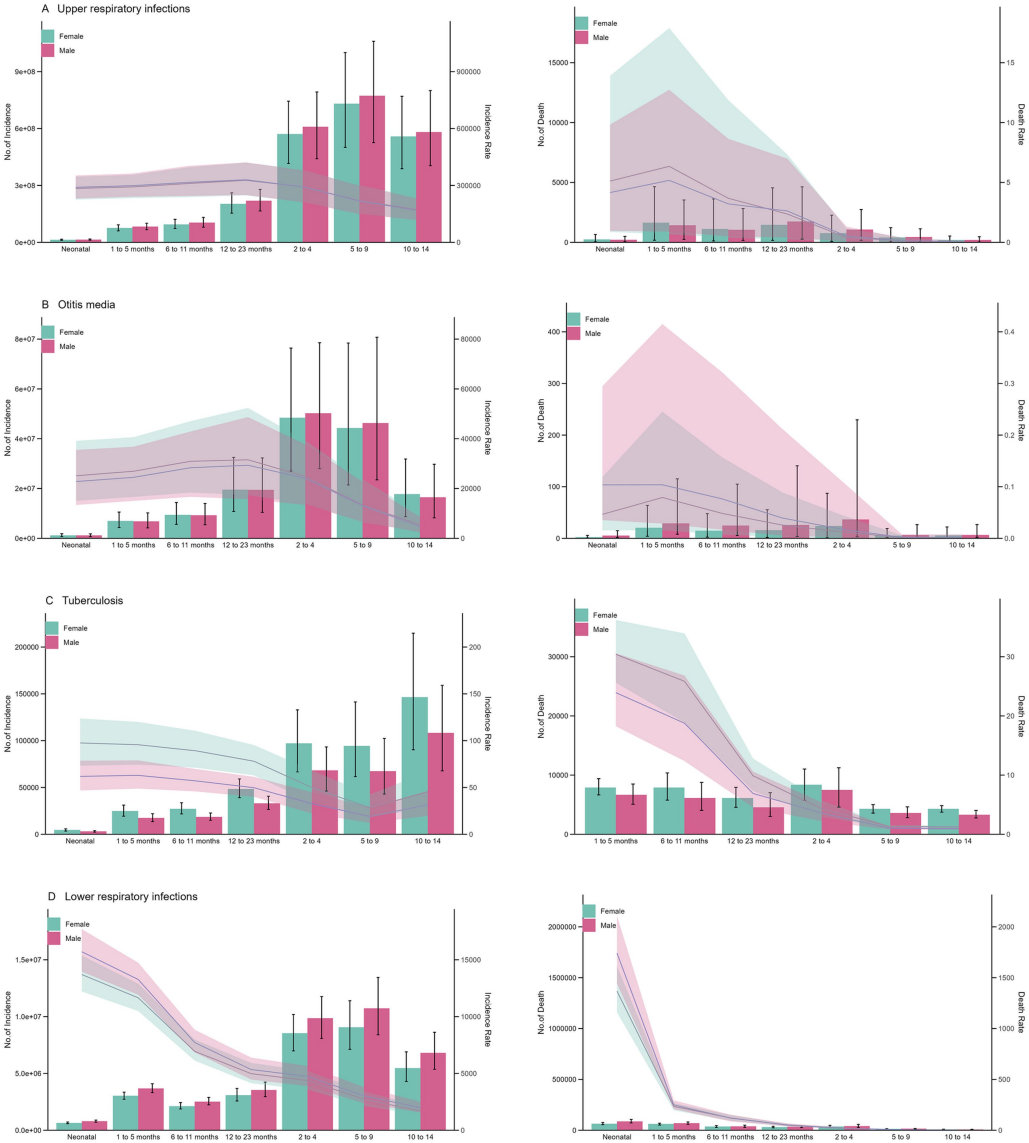
Global cases and deaths of RTIs, otitis media, and TB by age and sex in 2021. **(A)** Age-and sex-stratified case counts. **(B)** Age-specific incidence rates (bars: absolute numbers; error bars: 95% UI). **(C)** Sex ratio of mortality rates. **(D)** 95% uncertainty intervals for mortality ratios.

**Figure 4:**
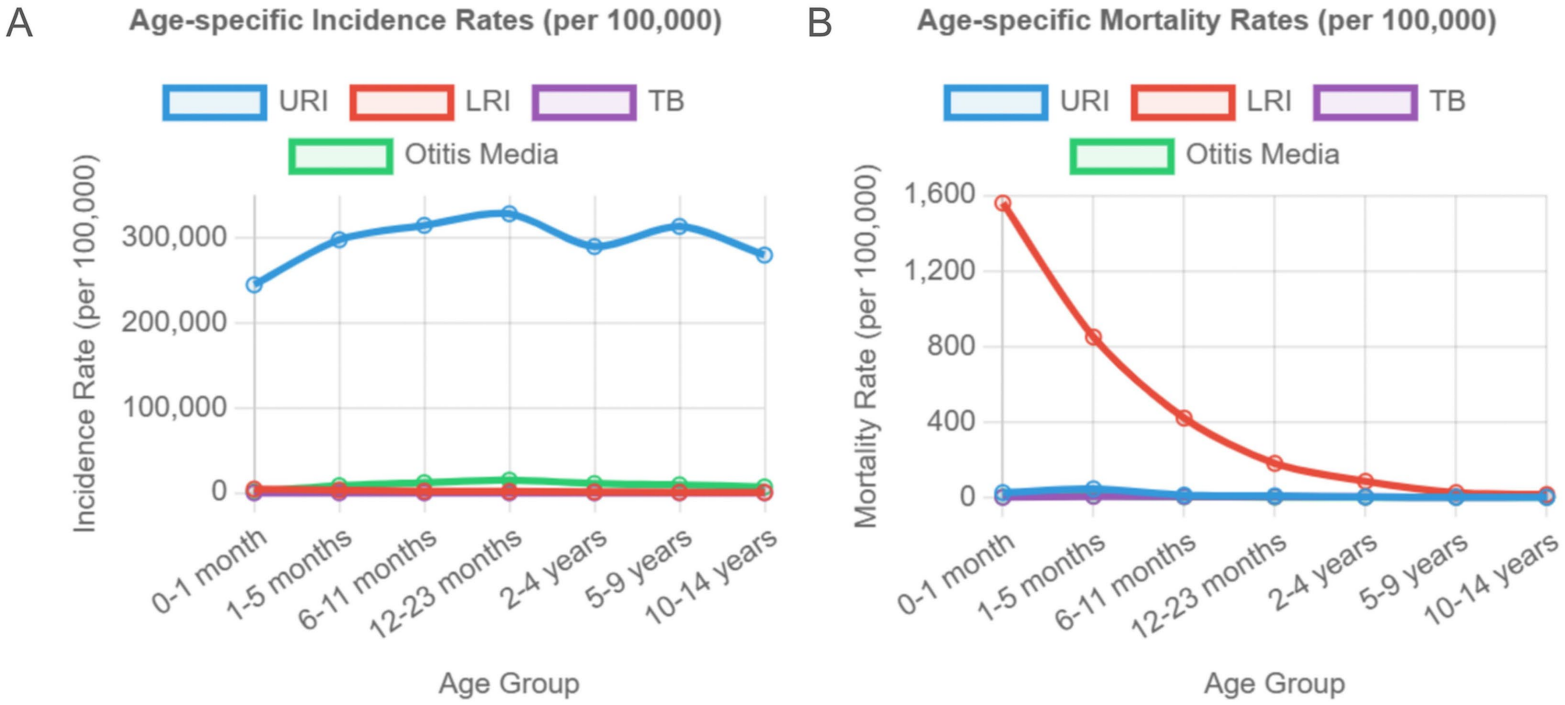
Age-specific Incidence and Mortality Patterns by Disease Type in 2021. **(A)** Age-specific incidence rates per 100,000 population and **(B)** age-specific mortality rates per 100,000 population for respiratory infections and tuberculosis in children aged 0–14 years in 2021. URI shows peak incidence in early childhood and school-age children, while LRI mortality is highest in neonates. TB incidence and mortality are highest in early infancy and decrease with age.

Age-stratified analysis showed peak URI incidence in 12–13-month-old infants (328,644/100 k) and 6–11-year-olds (313,772/100 k), with otitis media peaking in similar age groups (Figures 3A,B; Table 2). In contrast, the incidence of LRIs and TB was highest in the neonatal group (Figures 3C,D), and the incidence decreased significantly with the increase in age. The incidence of TB was lowest in the 5–9 years group at around 23.5/100 k (95% CI: 15.1, 35.6) (Supplementary Table 2). Although the incidence of each disease varied in different age groups, the mortality pattern was fairly similar. The mortality rates of URI and otitis media were highest in children aged 1–5 months, whereas the mortality rate of LRI was highest in the neonatal group, reaching 1,560.6/100 k (95% CI: 1,308.9, 1,835.7) (Supplementary Table 2; Figure 4). No burden of TB-related mortality was reported in the neonatal group (Figure 3). When stratified by sex, there was no significant difference in the incidence and mortality of URI, otitis media and LRI between boys and girls. However, the incidence and mortality of TB were significantly higher in boys than in girls (Figure 3).

**Table 2:** Top 5 risk factors for DALYs in children (1990 vs. 2021).

Risk factor	1990 DALYs rate (/100 k)	2021 DALYs rate (/100 k)	Rank change	PAF change (%)
Underweight children	3,798.0 (2,710.0–4,826.4)	680.6 (460.0–909.6)	— (1st → 1st)	−80.3 (76.1–84.8)
Household air pollution (HAP)	1,256.3 (1,012.5–1,523.8)	892.7 (710.5–1,089.4)	↑2 (5th → 3rd)	−29.0 (−32.5 – −25.1)
Low birth weight	987.4 (812.3–1,176.5)	765.3 (620.1–934.8)	↑1 (7th → 6th)	−22.5 (−26.8 – −17.9)
High temperature	452.1 (320.5–598.7)	510.2 (405.6–632.4)	↑4 (10th → 6th)	+12.8 (5.7–20.3)
Short gestational age	389.5 (298.6–501.4)	342.1 (265.3–431.7)	↑3 (11th → 8th)	−12.2 (−18.5 – −5.3)

PAF = Population attributable fraction; “↑n” indicates an increase in rank by *n* positions; DALYs = Disability-adjusted life years.

### Trends in the incidence and mortality of upper respiratory tract infection, otitis media, tuberculosis and lower respiratory tract infection

The incidence of URIs in children aged 0–14 years in 2021 was highest in the high-SDI regions at around 330,830.3/100 k (95% CI: 269,609.6, 397,982.4), significantly higher than the global average. The incidence was lowest in the low-SDI regions at 202,483.9/100 k (95% CI: 165,189.8, 244,270.0) (Figure 1A). Although the high-SDI regions had the highest incidence, the region had the lowest mortality rate, whereas the low-SDI regions had the heaviest disease burden and the highest mortality rate at around 2.036/100 k (95% CI: 0.192, 5.487). For children aged 0–14 years, except for URIs, the incidence and mortality of the diseases (otitis media, TB and LRIs) increased with the decrease in SDI value, and the low-SDI regions always ranked first (Figure 1).

Between 1990 and 2021, the incidence of otitis media in children aged 0–14 years in the high-SDI and global regions increased by 1.1% (95% CI: −0.85, 4.7) and 0.2% (95% CI: −1.5, 1.9), respectively. The incidence and mortality rates of other diseases in each SDI region showed a downward trend. The incidence rates of URI and otitis media in low-middle-SDI regions decreased the most at 6.1% (95% CI: 4.1, 8.2) and 5.9% (95% CI: 3.6, 7.7), respectively. The incidence rate of TB decreased the most in high-middle-SDI regions at around 75.5% (95% CI: 73.4, 77.5), and the incidence rate of LRI decreased the most in middle-SDI regions, by around 67.2% (95% CI: 65.3, 69.1). Except for TB, the mortality rates of all diseases decreased the most in high-middle-SDI regions. The largest decrease in TB mortality was in high-SDI regions at around 95.7% (95% CI: 94.6, 96.5), from 0.203/100 k (95% CI: 0.176, 0.235) in 1990 to 0.014/100 k (95% CI: 0.012, 0.016) in 2021 (Supplementary Table 3).

### Changes in risk factors associated with disability-adjusted life years of respiratory infections and tuberculosis in children aged 0–14 years

**Figure 5:**
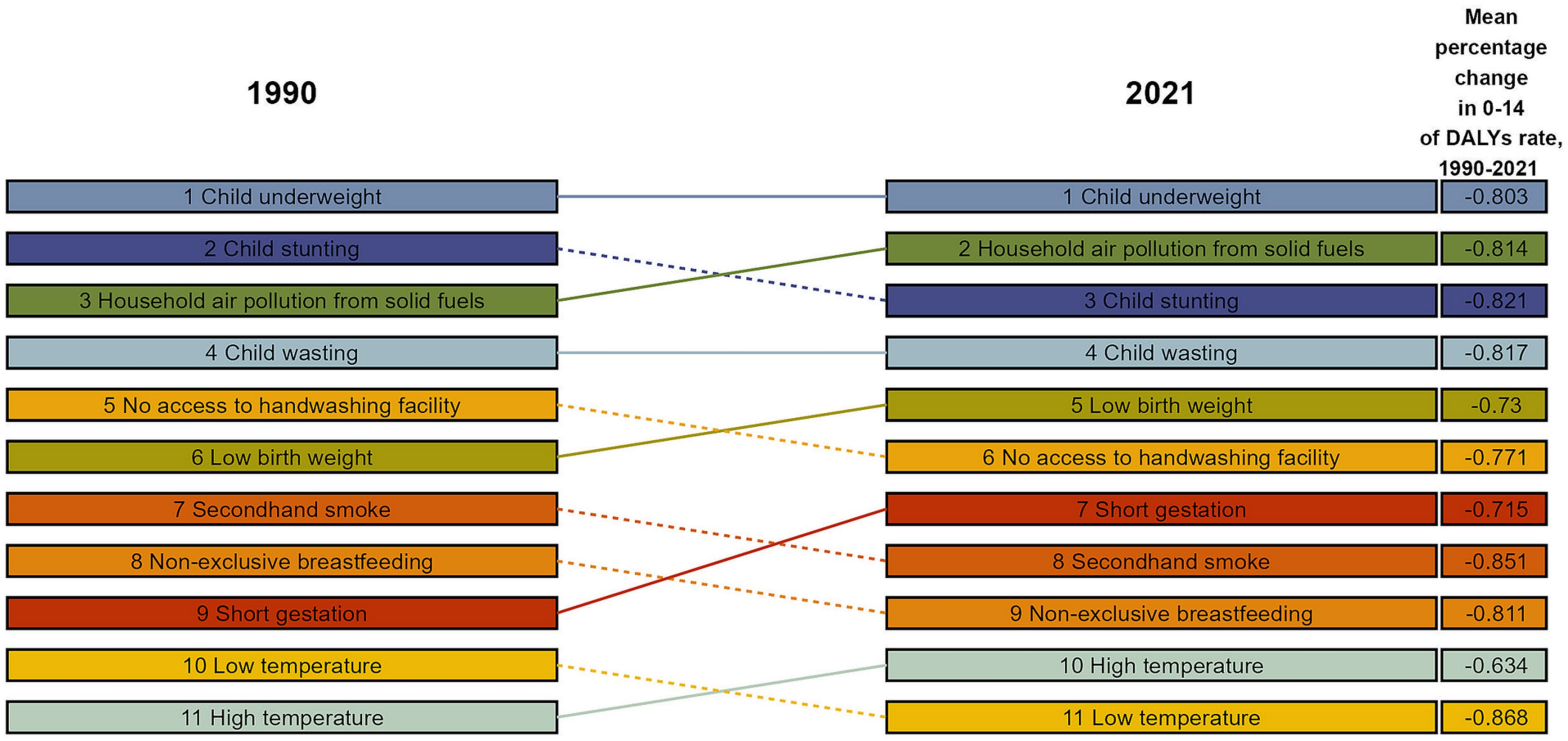
Percentage change in risk factors for DALYs of respiratory infections and tuberculosis in children aged 0–14 years, 1990–2021.

This study included 11 risk factors associated with DALYs of RTIs and TB in children aged 0–14 years: underweight children, stunting, HAP caused by solid fuels, child wasting, lack of handwashing facilities, low birth weight, second-hand smoke exposure, non-exclusive breastfeeding, short gestation period, low temperature and high temperature. Globally, underweight remains the leading risk factor, with DALY rates of 3,798/100 k (95% CI: 2,710.0, 4,826.4) in 1990 and 680.6/100 k (95% CI: 460.0, 909.6) in 2021, a decrease of 80.3% (95% CI: 76.1, 84.8%) (Figure 5; Supplementary Table 4). Although the DALY rates caused by all risk factors decreased between 1990 and 2021, the extent of the decrease varied. Except for low birth weight, the rankings of the remaining 10 risk factors changed significantly. Among the 11 risk factors associated with DALYs in 2021, HAP from solid fuels, low birth weight, short gestational age and high temperature ranked higher than in 1990, whereas the remaining risk factors ranked lower (Figure 5). The ranking of risk factors by SDI quintile showed a similar global trend. The leading risk factor in all SDI regions in both 1990 and 2021 was underweight, except for the middle-SDI region, where stunting was the leading risk factor in 1990 and underweight in 2021 (Supplementary Table 4).

### Key temporal patterns (1990–2021)

The 32-year analysis revealed divergent disease trajectories. While TB and LRI showed consistent global declines, SDI-stratified analysis revealed persistent inequalities: high-SDI regions achieved a 95.7% TB mortality reduction compared with 57.7% in low-SDI regions. The trends in URI diverged by development level, with high-SDI regions experiencing 15.2% increases versus 8.7% decreases in low-SDI areas, reflecting diagnostic and reporting disparities.

## Discussion

This study analysed the trends in the incidence, mortality and DALY rates of RTIs and TB among children in different SDI regions around the world between 1990 and 2021, and explored changes in gender, age and related risk factors. In 2021, the global incidence of URIs, otitis media and TB among children aged 0–14 years decreased considerably compared with 1990, but the overall incidence of RTIs and TB increased. The decline in the incidence of RTIs and TB among children may be related to the effectiveness of prevention and control measures (e.g., vaccination and improved hygiene habits) (7). However, new or inadequately controlled respiratory diseases (e.g., *Mycoplasma pneumoniae*, respiratory syncytial virus infection) are increasing. For example, the 2024 surveillance data of the Chinese Center for Disease Control and Prevention showed that the incidence of rhinovirus and *M. pneumoniae* increased in cases of acute RTIs in children aged 0–14 years (8). In addition, the reasons for the increase in the overall incidence of RTIs worldwide also include environmental factors such as population growth, changes in population structure, air pollution and climate change (9). The mutation of pathogens has led to a decrease in the effectiveness of existing vaccines and treatments, which may also be one of the factors leading to this trend (2). However, direct data on pathogen dynamics or healthcare access were not analysed, meaning these associations require validation in future studies.

The URI incidence peaked in 12–13-month-old infants (328,644/100 k) and 6–11-year-olds (313,772/100 k), likely due to immune naivety and school-based exposure (Figure 3A). Neonatal LRI mortality was highest (1,560.6/100 k), which was linked to airway immaturity (10). Studies have shown that childcare institutions and schools are important places for the transmission of respiratory pathogens, and close contact between children significantly increases the risk of infection (11). Regarding mortality, the mortality rate of respiratory infectious diseases is generally high in young children, especially in newborns and infants aged 1–5 months. The mortality rate of LRI is the highest in the neonatal group. The high mortality rate in young children may be related to factors such as immature immune system, insufficient medical resources and disease complications (10–12). Moreover, the anatomical characteristics of young children, such as smaller airway diameter and imperfect ciliary motility, increase the risk of respiratory secretion retention and secondary infection (13). There is no significant difference in the incidence and mortality of URI, otitis media and LRI between male and female children, but the incidence and mortality of TB are significantly higher in the former than in the latter. This may be related to sex hormones or gender bias in disease diagnosis and treatment (13, 14). Studies have shown that oestrogen has the effect of enhancing the anti-TB activity of macrophages, whereas androgens may inhibit Th1 immune response, which may be the biological basis for the higher susceptibility of male TB (15).

Therefore, in terms of prevention and control strategies, health education should be strengthened for children aged 12–13 months and 6–11 years, and these groups should be taught to develop good hygiene habits, such as washing hands frequently and avoiding touching the mouth and nose with the hands. In collective environments such as schools, ventilation should be strengthened and disinfection should be performed regularly. For newborns and young children, perinatal care should be strengthened and the health level of pregnant women should be improved to enhance the immunity of newborns. At the same time, more medical resources should be provided in economically underdeveloped areas to improve the diagnosis and treatment capabilities of primary medical institutions. In view of the higher incidence of TB in male children, TB screening and prevention work should be strengthened for these children to eliminate gender bias in the diagnosis and treatment of the disease.

High-SDI regions had the highest URI incidence but lowest mortality (0.014/100 k), likely due to diagnostic bias (16). Low-SDI regions had the highest TB mortality rate (2.036/100 k), which is linked to limited medical resources and poor hygiene. Although the incidence rate is lower in low-SDI areas, the disease burden and mortality rate are the highest in these areas, ranking first among all disease burdens. Low-SDI areas usually have a low level of economic development, insufficient public health infrastructure and scarce medical resources, and the local population lacks good hygiene habits and health awareness, which increases the risk of disease transmission. In addition, the vaccination rate in low-SDI areas is low, which may lead to poor results of preventive measures (16).

Between 1990 and 2021, the incidence of otitis media increased in both high-SDI regions and globally, which may be related to changes in the lifestyle of the population in the region, such as indoor pollution and increased exposure to allergens, potentially increasing the risk of the condition. In addition, with the advancement of medical technology, the diagnosis rate of otitis media may also have improved (16). The incidence and mortality rates of other diseases in various SDI regions have declined. The mortality rate of TB in high-SDI regions has dropped the most, reaching 95.7%, which may be due to the regions’ investment and efforts in TB prevention and control, including vaccination, early diagnosis and effective treatment (16). Notably, the rising otitis media incidence in high-SDI regions may reflect diagnostic advancements. For example, otoacoustic emission testing, introduced post-2000, improved detection of occult otitis media by around 30% (3), whereas underdiagnosis persists in low-SDI areas due to resource constraints. Additionally, pneumococcal conjugate vaccine rollout in high-SDI regions since 2010 reduced bacterial pneumonia mortality but may have increased non-vaccine pathogen infections (e.g., *M. pneumoniae*) via competitive exclusion (4). These temporal disparities in diagnostics and interventions require cautious interpretation of trends.

The results of this study show that although underweight was the leading risk factor for DALYs of RTIs and TB between 1990 and 2021, the DALY rate of children has dropped by 80.3%, reflecting the significant effectiveness of global nutrition interventions (e.g., food aid and vitamin A supplementation programmes) (17, 18). However, underweight remained the leading risk factor in 2021, suggesting persistent nutrition problems in low-income regions (especially low-SDI countries). The rankings of HAP and high temperatures caused by solid fuels have risen, which is consistent with current research. Although the global use of clean energy has increased, solid fuels are still relied upon in sub-Saharan Africa and South Asia, and the association between HAP and childhood pneumonia is particularly significant (19). Climate change has exacerbated extreme heat events, which may indirectly increase the risk of RTIs by promoting the spread of pathogens and weakening children’s immunity (e.g., dehydration) (20). Related studies in tropical regions have observed a positive correlation between high temperature and pneumonia hospitalisation rates (21). The ranking of low birth weight has not dropped significantly, suggesting that its intervention is more difficult. Recent studies have shown that the long-term impact of low birth weight on respiratory diseases may involve epigenetic regulation (22), meaning multi-dimensional intervention is needed. In the changes of risk factors in SDI zoning, the middle-SDI regions had stunting as the top risk factor in 1990, but this was replaced by underweight in 2021. This may reflect the contradictions in the areas’ nutritional transition period: in the early stages of economic development, increased calorie intake may prioritise improving height development, but insufficient dietary quality leads to persistent underweight (22). In addition, public health resources may be more focused on acute malnutrition (wasting) and may underestimate the long-term impact of chronic malnutrition (23). Notably, risk factors could interact synergistically. For example, malnutrition exacerbates mucosal damage from HAP: underweight children exposed to HAP have 2.3-fold higher pneumonia incidence than normal-weight children (5). Additionally, high temperatures may impair ciliary clearance via dehydration, compounding the risk of neonatal LRI mortality with low birth weight (6). This study did not quantify such interactions, warranting future investigation via structural equation modelling.

This study revealed three major contradictions in the global burden of childhood respiratory diseases. Although vaccination has reduced the incidence of specific infections, population growth, pathogen variation and environmental degradation continue to lead to an increase in the overall disease burden. There are significant differences in SDI stratification. Low-SDI regions are limited in terms of medical resources and clean energy, and urgently need to strengthen nutritional intervention and health facility construction. Risk factors are changing dynamically. Traditional risks (e.g., malnutrition) remain dominant, while emerging risks (e.g., climate change) need to be included in multi-sector collaborative governance.

However, this study has certain limitations. First, GBD data are based on model estimation, which may lead to underreporting in low-SDI areas because the health surveillance systems in these areas are relatively weak, and data collection and reporting may be biased. Notably, the study assumes homogeneity in exposure and data completeness across SDI groups, a premise that may introduce systematic bias. For example, low-SDI regions often exhibit fragmented health surveillance, potentially underreporting mild infections while overreporting severe cases (1). Additionally, spatial interpolation methods for data-scarce areas (e.g., inference from neighbouring high-SDI regions) might misestimate true disease burdens in resource-constrained settings (2). Second, the representativeness of the sample may be limited, especially in low-resource areas, where some cases may not be included in the statistics due to insufficient medical facilities and high population mobility. In addition, there may be inconsistencies in data collection methods between different regions and years, such as differences in diagnostic criteria, reporting mechanisms and statistical calibres, which may affect the cross-regional and cross-temporal comparability of the results. Finally, this study did not conduct an in-depth analysis of the interactions between risk factors, such as the synergistic effects between malnutrition and air pollution and climate change, which may underestimate the actual impact of certain risk factors.

Future research should focus on disease surveillance and data quality improvement in low-SDI areas and improve the accuracy and completeness of data collection by strengthening the construction of primary medical facilities, training health workers and using mobile medical technologies (e.g., electronic health records and remote monitoring). At the same time, it is necessary to explore the interaction of multiple risk factors (e.g., malnutrition, air pollution and pathogen exposure) and their cumulative effects on the disease burden, and to use multivariate analysis methods (e.g., structural equation models and causal inference methods) to reveal their complex relationships. Finally, addressing the global burden of childhood respiratory diseases requires cross-border collaboration, and future research should explore how to establish more effective international cooperation mechanisms – including data sharing platforms, joint research projects and cross-regional policy coordination – and strengthen technology transfer and capacity building in low-SDI regions to narrow the global health gap. By clarifying these research directions, this study provides a scientific basis for the future prevention and control of global childhood respiratory diseases, and calls for multidisciplinary and multi-departmental collaborative efforts to promote progress in this field.

## Conclusion

This study analysed the changes in the global incidence, mortality and disease burden of RTIs and TB in children (aged 0–14) between 1990 and 2021. It was found that although vaccination and improved hygiene measures have achieved certain results, the overall disease burden is still rising due to pathogen variation, environmental degradation and population growth. Low-SDI regions continue to face a high disease burden due to lack of resources, and it is crucial to strengthen nutritional intervention and health facility construction. Underweight is the main risk factor for RTIs and TB in children worldwide; however, with the improvement of nutritional intervention, the associated DALY rate has dropped significantly. Emerging risks such as climate change and air pollution are also gradually increasing, and their impact on children’s health needs to be considered. Medical resources and prevention and control measures in high-SDI regions help reduce mortality, demonstrating the importance of early diagnosis and treatment. In short, the global burden of respiratory diseases in children faces multiple challenges, and it is necessary to strengthen international cooperation and take precise intervention measures, especially in low-SDI regions, to improve public health and nutrition levels.

## Data Availability

The original contributions presented in the study are included in the article/Supplementary material, further inquiries can be directed to the corresponding author.

## References

[1] Troeger C Blacker B Khalil I Khalil IA Rao PC Cao J. Estimates of the global, regional, and national morbidity, mortality, and aetiologies of lower respiratory infections in 195 countries, 1990–2016: a systematic analysis for the global burden of disease study 2016. Lancet Infect Dis. (2018) 18:1191–210. doi: 10.1016/S1473-3099(18)30310-4, PMID: 30243584 PMC6202443

[2] Zar HJ Ferkol TW. The global burden of respiratory disease—impact on child health. Pediatr Pulmonol. (2014) 49:430–434. doi: 10.1002/ppul.23030, PMID: 24610581

[3] Troeger C Forouzanfar M Rao PC Khalil I Brown A Swartz S. Estimates of the global, regional, and national morbidity, mortality, and aetiologies of lower respiratory tract infections in 195 countries: a systematic analysis for the global burden of disease study 2015. Lancet Infect Dis. (2017) 17:1133–61. doi: 10.1016/S1473-3099(17)30396-1 28843578 PMC5666185

[4] Dodd PJ Yuen CM Sismanidis C Seddon JA Jenkins HE. The global burden of tuberculosis mortality in children: a mathematical modelling study. Lancet Glob Health. (2017) 5:e898–906. doi: 10.1016/S2214-109X(17)30289-9 28807188 PMC5556253

[5] Seddon J Shingadia D. Epidemiology and disease burden of tuberculosis in children: a global perspective. Infect Drug Resist. (2014) 7:153–65. doi: 10.2147/IDR.S45090, PMID: 24971023 PMC4069045

[6] World Health Organization [EB/OL]. (2024). Available online at: https://www.who.int/news-room/fact-sheets/detail/malnutrition

[7] Yang H Ruan X Li W Xiong J Zheng Y. Global, regional, and national burden of tuberculosis and attributable risk factors for 204 countries and territories, 1990–2021: a systematic analysis for the global burden of diseases 2021 study. BMC Public Health. (2024) 24:3111. doi: 10.1186/s12889-024-20664-w 39529028 PMC11552311

[8] Wang Y Chen J Feng W Ding X Han R Anning MA. Trends in the burden of lower respiratory tract infections among children aged <5 years in the world, high SDI countries and China from 1990 to 2019. Chin J Public Health. (2024) 40:840–8. doi: 10.11847/zgggws1143671

[9] World Health Organization. Global strategy for tuberculosis research and innovation. Geneva: World Health Organization (2020).

[10] Xin CJ Cao LJ Lü LG. Analysis of risk factors for recurrent respiratory tract infections in children. J Tubercul Lung Dis. (2022) 3:300–4. doi: 10.19983/j.issn.2096-8493.20220060

[11] Chen J Liu SY Zhou LL. Analysis of common pathogens of respiratory tract infections in children in Lishui City. Prev Med. (2021) 33:529–31. doi: 10.19485/j.cnki.issn2096-5087.2021.05.025

[12] Wilkes C Bava M Graham H Duke T ARI Review group. What are the risk factors for death among children with pneumonia in low-and middle-income countries? A systematic review. J Glob Health. (2023) 13:13. doi: 10.7189/jogh.13.05003, PMID: 36825608 PMC9951126

[13] Chinese Medical Association Pediatrics Branch Respiratory Group Difficult and Rare Diseases Collaboration Group, National Clinical Research Center for Respiratory Diseases, Editorial Board of Chinese Journal of Practical Pediatrics. Expert consensus on the diagnosis and treatment of primary ciliary dyskinesia in children. Chin J Pract Pediatr. (2019) 33:94–9. doi: 10.3760/cma.j.issn.2095-428X.2018.02.004

[14] Ingersoll M. Sex differences shape the response to infectious diseases. PLoS Pathog. (2017) 13:e1006688. doi: 10.1371/journal.ppat.1006688, PMID: 29284060 PMC5746274

[15] Kissick HT Sanda MG Dunn LK Pellegrini KL On ST Noel JK. Androgens alter T-cell immunity by inhibiting T-helper 1 differentiation. Proc Natl Acad Sci USA. (2014) 111:9887–92. doi: 10.1073/pnas.1402468111, PMID: 24958858 PMC4103356

[16] Chen C You Y Du Y Zhou W Jiang D Cao K. Global epidemiological trends in the incidence and deaths of acute respiratory infections from 1990 to 2021. Heliyon. (2024) 10:e35841. doi: 10.1016/j.heliyon.2024.e35841, PMID: 39224281 PMC11367038

[17] Scott N Delport D Hainsworth S Pearson R Morgan C Huang S. Ending malnutrition in all its forms requires scaling up proven nutrition interventions and much more: a 129-country analysis. BMC Med. (2020) 18:356. doi: 10.1186/s12916-020-01786-5, PMID: 33183301 PMC7661178

[18] Bhutta Z Baker S. Premature abandonment of global vitamin a supplementation programmes is not prudent. Int J Epidemiol. (2015) 44:297–9. doi: 10.1093/ije/dyu274, PMID: 25638819

[19] Gordon SB Bruce NG Grigg J Hibberd PL Kurmi OP Lam KBH. Respiratory risks from household air pollution in low and middle income countries. Lancet Respir Med. (2014) 2:823–60. doi: 10.1016/S2213-2600(14)70168-7, PMID: 25193349 PMC5068561

[20] Chitre SD Crews CM Tessema MT Plėštytė-Būtienė I Coffee M Richardson ET. The impact of anthropogenic climate change on pediatric viral diseases. Pediatr Res. (2024) 95:496–507. doi: 10.1038/s41390-023-02929-z, PMID: 38057578 PMC10872406

[21] He Q Liu Y Yin P Gao Y Kan H Zhou M. Differentiating the impacts of ambient temperature on pneumonia mortality of various infectious causes: a nationwide, individual-level, case-crossover study. EBioMedicine. (2023) 98. doi: 10.1016/j.ebiom.2023.104854, PMID: 38251462 PMC10628343

[22] Hopkinson NS Bush A Allinson JP Faner R Zar HJ Agustí A. Early life exposures and the development of chronic obstructive pulmonary disease across the life course. Am J Respir Crit Care Med. (2024) 210:572–80. doi: 10.1164/rccm.202402-0432PP, PMID: 38861321

[23] Victora CG Christian P Vidaletti LP Gatica-Domínguez G Menon P Black RE. Revisiting maternal and child undernutrition in low-income and middle-income countries: variable progress towards an unfinished agenda. Lancet. (2021) 397:1388–99. doi: 10.1016/S0140-6736(21)00394-9, PMID: 33691094 PMC7613170

